# Network Pharmacology Approach and Experimental Verification to Explore the Anti-NSCLC Mechanism of Grifolic Acid

**DOI:** 10.3390/ijms26020629

**Published:** 2025-01-13

**Authors:** Xiangdan Cuan, Jinxian Wang, Yue Zhao, Jingyun Yan, Jun Sheng, Yanping Huang

**Affiliations:** 1 Key Laboratory of Pu-Er Tea Science, Ministry of Education, Yunnan Agricultural University, Heilongtan, North of Kunming, Kunming 650201, China; cuanxiangdan@163.com (X.C.); 18187055749@163.com (J.W.); zhao20201414@163.com (Y.Z.); yan604100899@126.com (J.Y.); 2 College of Food Science and Technology, Yunnan Agricultural University, Heilongtan, North of Kunming, Kunming 650201, China; 3 College of Science, Yunnan Agricultural University, Heilongtan, North of Kunming, Kunming 650201, China

**Keywords:** lung cancer, grifolic acid, erlotinib, EGFR, combination-therapy

## Abstract

Lung cancer is the leading cause of cancer-related death. Non–small cell lung cancer (NSCLC) accounts for 85% of all lung cancers and over 60% express wild-type EGFR (WT-EGFR); however, EGFR tyrosine kinase inhibitors (TKIs) have limited effect in most patients with WT-EGFR tumors. In this study, we applied network pharmacology screening and MTT screening of bioactive compounds to obtain one novel grifolic acid that may inhibit NSCLC through the EGFR-ERK1/2 pathway. Through the PPI network and machine learning, we identified two hub genes, EGFR and AKT1, as potential therapeutic targets. Molecular docking confirmed that the grifolic acid could effectively bind to the key target, EGFR. Using the NSCLC cell line NCI-H1781 as an in vitro model, we evaluated the effect of the drugs’ combination on viability, apoptosis, and clonogenicity capacity. In vitro studies showed that combined treatment decreased cell viability, increased activation PARP, and caused cell cycle redistribution and significantly greater inhibition of pEGFR and pAKT. This study not only provides new insights into the mechanism of grifolic acid against NSCLC but also important information and new research ideas for the discovery of anti-NSCLC compounds from natural products.

## 1. Introduction

Lung cancer, including non-small-cell lung cancer and small-cell lung cancer, is the most common leading cancer-related death in the world. The epidermal growth factor receptor (EGFR) is considered a molecular target for the treatment of cancer due to its abnormal expression or activation in a variety of cancer cells, especially non-small-cell lung cancer (NSCLC), which accounts for 84% of lung tumors [1,2]. EGFR is a member of the epidermal growth factor receptor (HER) family. All EGFR family members have a similar molecular structure, which includes extracellular ligand binding domains, a single transmembrane helix, and an intracellular domain [3]. EGFR ligands are members of the EGF family of peptide growth factors and include amphiregulin (AR), epiregulin (EPR), transforming growth factor alpha (TGFα), heparin-binding EGF-like growth factor (HB-EGF), epigen (EPG), betacellulin (BTC), neuregulin 2 beta (NRG2β), and EGF [4,5]. EGFR is activated by ligand binding, triggering receptor dimerization, activation of kinase activity, and intracellular signaling [6].

The EGFR autocrine pathway plays a crucial role in human cancer since it contributes to a number of highly relevant processes in tumor development and progression, including cell proliferation, regulation of apoptotic cell death, angiogenesis, and metastatic spread. Among a variety of approaches used to target EGFR signaling, EGFR blocking monoclonal antibodies and small molecular of the EGFR tyrosine kinase inhibitors (TKI) have been successfully developed [7]. EGFR monoclonal antibodies (cetuximab and panitumumab) that target the EGFR are approved for treatment of colorectal, head, and neck cancers, whereas its development in NSCLC has not taken its place in routine clinical practice, because of limited clinical benefit despite statistically significant Phase III trials [8]. Erlotinib (Tarceva, OSI Pharmaceuticals, Melville, NY, USA) inhibits EGFR tyrosine kinase autophosphorylation by inhibition of the intracellular domain. On 18 November 2004, the FDA granted erlotinib regular approval for treatment of patients with locally advanced or metastatic NSCLC after failure of at least one prior chemotherapy regimen [9]. However, clinical trials have revealed that erlotinib provides a durable benefit in patients whose tumors harbor activating mutations of EGFR [10]. Erlotinib is the first generation of EGFR-TKIs that was designed to treat tumors expressing EGFR, but it brings more benefits to cells harboring than L858R mutation and exon 19 deletions [11,12]. Unfortunately, although first-generation EGFR-TKIs have proven effective in the early treatment, almost all tumors invariably acquire resistance to these TKIs within 9 to 13 months [11,12]. The most common mechanism of TKI resistance is a second-site mutation (T790M) in the EGFR kinase domain, which accounts for more than 50% of these cases. In addition, the activation of signaling molecules, including MET, HER2 amplification, and PIK3CA and KRAS mutations, also largely contributed to the acquired resistance to EGFR TKIs [13,14,15]. Although a series of EGFR-TKIs have been developed for overcoming resistance, the remaining resistance mechanisms to this TKI are largely unknown [16,17]. Thus, developing novel therapies against resistance is critical.

Grifolic acid is a phenolic compound that was first extracted from the mushroom *Albatrellus confluens*, and it was reported as an agonist of the free fatty acid receptor (FFAR4). FFAR4 is expressed in macrophages and mediates the anti-inflammatory effects of *n3* unsaturated free fatty acids [18,19,20]. However, the effect of grifolic acid on EGFR signaling and its mechanism by which grifolic acid mediates the progression of NSCLC have not been researched at all.

To investigate the therapeutic strategies for treating patients with overexpression of EGFR, we performed drug screening and found grifolic acid to be a promising candidate against WT-EGFR according to the in vitro assays. Our current studies suggest that the combination of grifolic acid and erlotinib could inhibit the phosphorylation of EGFR and downstream ERK and AKT signaling, and retard G0 to S phase transitions, resulting in the activation of apoptosis. Finally, our data demonstrated that grifolic acid has potential as a therapeutic agent for WT-EGFR. It is tempting to speculate that a combination of EGFR TKI and grifolic acid could serve as a novel combinatory therapy for WT-EGFR NSCLC.

## 2. Results

### 2.1. Screening for EGFR Targeting Compounds

**Figure 1:**
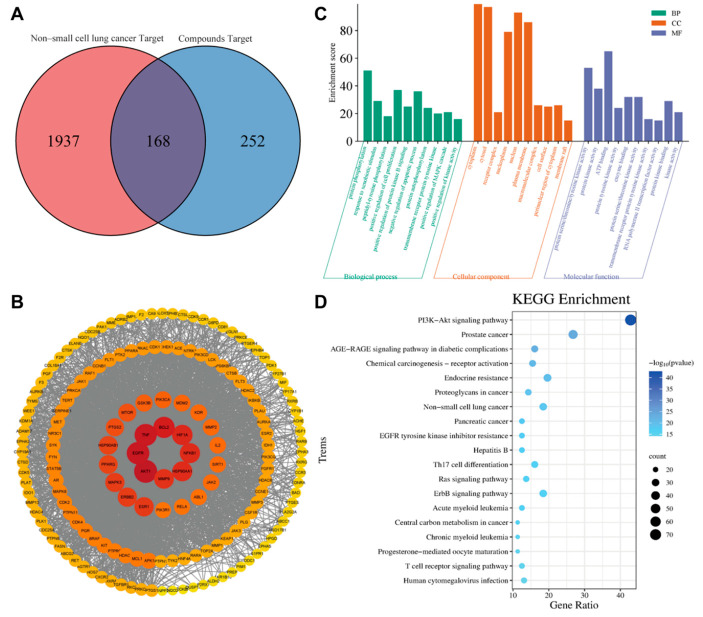
Network pharmacological analysis. (**A**) Wayne diagram of the intersection of compounds and non-small cell lung cancer targets. (**B**) Network of protein–protein interaction. (**C**) GO enrichment map and (**D**) KEGG enrichment analysis bubble map of the intersection targets. The larger the node area and the darker the color in the figure, the greater the influence.

In our preliminary study, we screened an ~1200 compound library using purified EGFR extracellula. The biochemical screen was carried out using 10 μM of the Biacore S200 SPR system to obtain the corresponding response values greater than 10 and estimate the binding of EGFR. Based on these corresponding response values, we screened the top 17 natural compounds based on their relative content (Supporting Information, Table S1). Furthermore, the compounds were searched for relevant targets using the SwissTargetPrediction database, and disease target networks were constructed for each compound using Cytoscape v 3.9.1. As shown in Figure 1A, the intersection of compounds targets and NSCLC disease targets was taken, and Venn diagram was drawn by R software to obtain 168 intersection targets. The PPI network of potential targets of compounds against NSCLC was constructed using the String platform. The protein–protein interaction networks suggested that 167 proteins and 2992 interactions (edges) could potentially interact in the intersection targets between compound and NSCLC, and the darker color represents a more critical influence of the target in the protein interaction relationship. As shown in Figure 1B, EGFR, AKT1, TNF, BCL2, HIF1A, NFKB1, HSP90AA1, and MMP9 were discovered to be very important in the network by analyzing network topology parameters.

Meanwhile, the results indicate that response to protein phosphorylation, response to negative regulation of apoptotic process, and positive regulation of cell proliferation were tightly related to biological processes. The main disease-related terms in cellular components consisted of cytoplasm, cytosol, and nucleus. Concerning molecular functions, more significant enrichment was found in protein serine/threonine/tyrosine kinase activity, ATP binding, and protein kinase activity (Figure 1C). The results of KEGG functional enrichment analysis showed that the active components affected NSCLC mainly through PI3K-Akt signaling pathway and the ErbB signaling pathway, indicating that compounds might play a major role in the treatment of NSCLC through the pathways (Figure 1D). The GO and KEGG enrichment analysis of compounds intervention NSCLC-related targets was carried out with R software, and the results were visualized (Figure 1C,D). In the KEGG pathway analyses, the results demonstrated that the PI3K-Akt signaling pathway and the ErbB signaling pathway were the key pathway in the NSCLC (Figure 1D).

### 2.2. In the Presence of EGF, the Combination of Grifolic Acid and Erlotinib Reduces the Viability of NCI-H1781 Cells

According to network pharmacology findings, EGFR and AKT1, which were the top-ranked proteins in the PPI network, might play critical roles in the therapeutic effects of natural compounds against NSCLC. To verify the treatment effect of natural compound on the NSCLC, we initially examined four NSCLC cell lines (NCI-H441, NCI-H1781, NCI-H1975 and A431), which express comparable amounts of total EGFR protein and activated EGFR at basal level. Hence, a series of four kinase inhibitors was screened to identify whether 15 natural compound combination therapies could more effectively target EGFR NSCLC cells compared to single agents. Next, treatment of NCI-H1781 cells with the combination grifolic acid/erlotinib decreased cell viability to a larger extent than each treatment alone. This result shows that the combination of grifolic acid/erlotinib is significantly more efficient to reduce cell proliferation and viability compared to the monotherapies in vitro.

**Figure 2:**
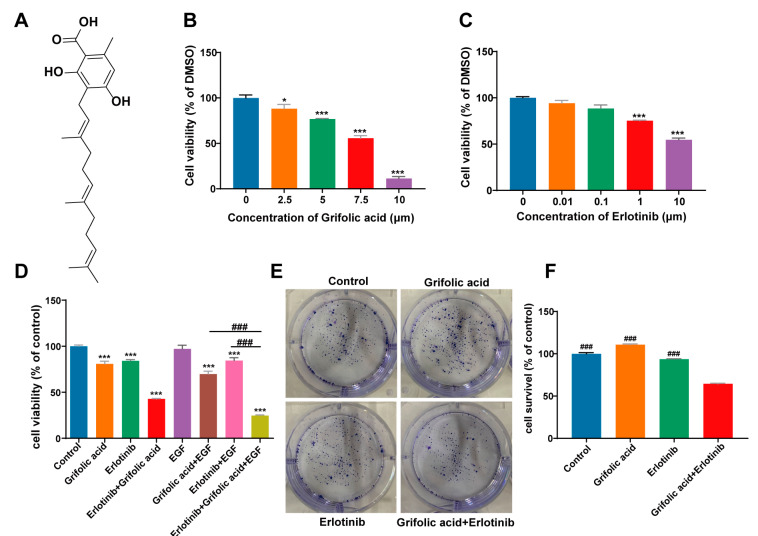
Cell viability assay. (**A**) Chemical structure of grifolic acid. (**B**) The NCI-H1781 cells were treated with grifolic acid 2.5, 5, 7.5, 10 μM. (**C**) NCI-H1781 cells were treated with erlotinib at concentrations of 0.01, 0.1, 1, and 10 μM. MTT assay was performed to determine cell viability. (**D**) In the presence of EGF (10 ng/mL), NCI-H1781 cells were treated with grifolic acid (5 μM) or combined with erlotinib (1 μM) for 48 h. (**E**) The effect of grifolic acid on anchorage-dependent cell growth of NCI-H1781 cells. (**F**) Quantification of clonogenic formation. The results were analyzed by one-way ANOVA with a significant difference versus the combination-therapy group, ^###^ *p* < 0.001. There were significant difference versus control, * 0.01 ≤ *p* < 0.05, *** *p* < 0.001. The experiments were repeated at least three times.

The chemical structure of grifolic acid was presented in Figure 1A. First, we evaluated the effects of grifolic acid on the proliferation of NCI-H1781 cells. We conducted MTT assays and found that grifolic acid significantly inhibited the viability of NCI-H1781 cells mostly in a dose-dependent manner (Figure 1B). The IC_50_ values of grifolic acid NCI-H1781 cells were 6.7 ± 0.413 μmol/L. Similarly, the IC_50_ value of NCI-H1781 to erlotinib was approximately 10 μM (Figure 2C). Next, we combined grifolic acid with erlotinib and investigated the efficacy in NCI-H1781 cells by performing the MTT assay. Grifolic acid alone and erlotinib alone decreased cell viability up to 80% at concentrations of 5 μM and 85% at 1 μM, respectively (Figure 2D; lane 2 and lane 3, Figure 2D). Moreover, the combination of grifolic acid and erlotinib further decreased the survival percentage compared to grifolic acid and erlotinib alone. And in the presence of EGF (10 ng/mL), grifolic acid and erlotinib decreased cell viability up to 30.23% and 15.58%, respectively. The combination of grifolic acid and erlotinib further decreased the survival percentage by 75.26%. These results indicate that the inhibitory effect of the combined action is more obvious than the inhibitory effect of single use. Consistent with the MTT data, the combination of grifolic acid and erlotinib decreased the colony formation of NCI-H1781 (*p* < 0.001, Figure 2E,F).

### 2.3. Molecular Docking Analysis

**Figure 3:**
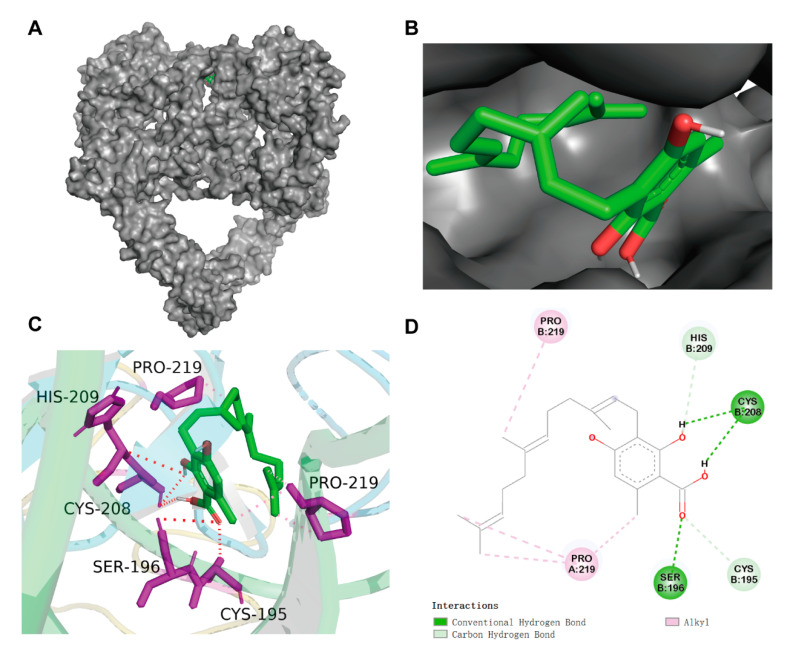
Molecular modeling of grifolic acid–EGFR. (**A**) Grifolic acid-docked EGFR structure. (**B**) Zoom-in of the grifolic acid binding pocket. (**C**) The results of grifolic acid docked to EGFR. Grifolic acid is depicted as sticks (green), residues as sticks (magentas), hydrogen bonds as dashed red lines, and hydrophobic interactions as dashed pink lines. (**D**) 2D model of grifolic acid interaction with EGFR.

Molecular docking was performed to investigate potential binding modes and the reliability of the interactions between the grifolic acid and EGFR. Grifolic acid was docked with EGFR using AutoDock Vina. The binding mode of grifolic acid at the EGFR pocket is shown in Figure 3A,B. The best binding energy of grifolic acid–EGFR was—4.1 kcal/mol. As shown in Figure 3C,D, Table 1, Cys-208 (d = 4.8 Å, 4.3 Å,) and Ser-196 (d = 3.9 Å) formed three hydrogen bonds. Cys-195 (d = 4.7 Å) and His-209 (d = 4.9 Å) formed two carbon hydrogen bonds. Moreover, the residues of Pro-219 (Chain A, Chain B) were involved in hydrophobic interaction formation. These data indicate that hydrogen bonding and hydrophobic interaction may play the main role in the grifolic acid–EGFR complex.

### 2.4. In the Presence of EGF, Grifolic Acid Combined with Erlotinib Inhibited Phosphorylation of EGFR and Downstream Signaling Proteins in NCI-H1781 Cells

**Figure 4:**
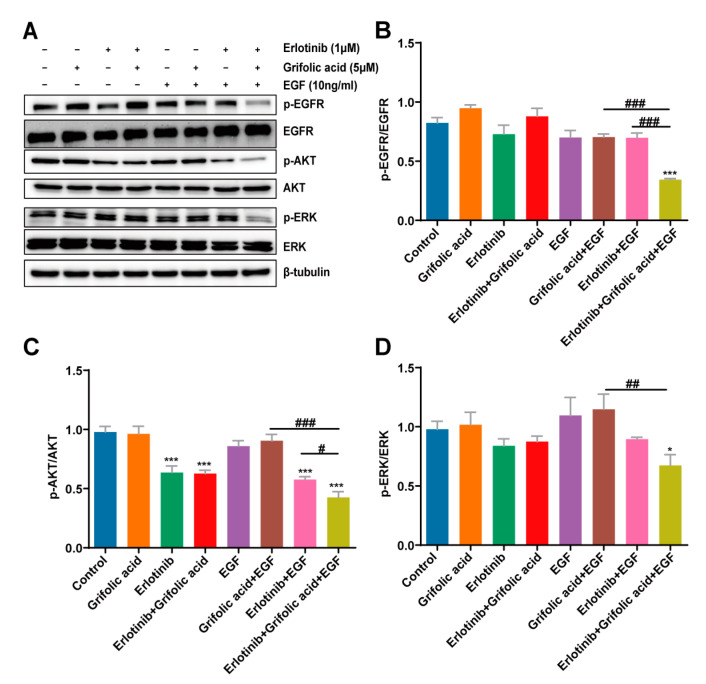
Expression of EGFR and downstream related proteins. The NCI-H1781 cells were treated with erlotinib (1 μM) for 1 h, then treated with grifolic acid (5 μM) and EGF (10 ng/mL) for 30 min. EGFR phosphorylation and downstream signaling were assessed by immunoblot. β-tubulin was used as a loading control. The experiments were repeated at least three times. Quantitative analysis (**B**–**D**) of (**A**). The WB gels in Figure 4 were the cropping of the full-length gels. The full-length gels are presented in Supplementary Figure S2. The results were analyzed by one-way ANOVA with a significant difference versus combination-therapy group, ^#^ 0.01 ≤ *p* < 0.05, ^##^ 0.001 ≤ *p* < 0.01, ^###^ *p* < 0.001. There was a significant difference versus control, * 0.01 ≤ *p* < 0.05, *** *p* < 0.001.

To further investigate the mechanism of growth inhibition, we performed Western blotting to analyze the “EGFR signaling pathway” in NCI-H1781 cells, as shown in (Figure 4A–D). The results show that in the presence of EGF, erlotinib in combination with grifolic acid induced greater inhibition of pEGFR, pERK, and pAKT than erlotinib alone. Together, these data indicate that the combination of grifolic acid and erlotinib inhibits EGFR pathways and controls EGFR-mediated phenotypes, including tumorigenesis and proliferation.

### 2.5. In the Presence of EGF, Grifolic Acid Combined with Erlotinib Induces Apoptosis in NCI-H1781 Cells

**Figure 5:**
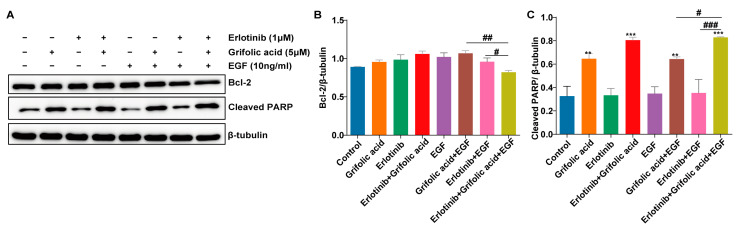
Expression of apoptosis-related proteins. The NCI-H1781 cells were treated with erlotinib (1 μM), grifolic acid (5 μM), and EGF (10 ng/mL) for 24 h. Apoptosis-related proteins were assessed by immunoblot. β-tubulin was used as a loading control. The experiments were repeated at least three times. Quantitative analysis (**B**,**C**) of (**A**). The WB gels in Figure 5 were the cropping of the full-length gels. The full-length gels are presented in Supplementary Figure S3. The results were analyzed by one-way ANOVA with a significant difference versus combination-therapy group, ^#^ 0.01 ≤ *p* < 0.05, ^##^ 0.001 ≤ *p* < 0.01, ^###^ *p* < 0.001. There was a significant difference versus control, ** 0.001 ≤ *p* < 0.01, *** *p* < 0.001.

Next, we determined the expression levels of Bcl-2 and cleaved PARP, which are the hallmarks of apoptosis and play crucial roles in this cellular process. Compared to the untreated control and the individual drugs, grifolic acid plus erlotinib induced a significant increase in cleaved PARP and a decrease in Bcl-2 (Figure 5A–C). The results show that the inhibitory ability of grifolic acid combined with erlotinib on NCI-H1781 cells may be achieved by inhibiting the expression of anti-apoptotic proteins and promoting the expression of pro-apoptotic proteins in the presence of EGF.

### 2.6. In the Presence of EGF, Grifolic Acid Combined with Erlotinib Reduces the Expression of Cycle Marker Proteins in NCI-H1781 Cells

**Figure 6:**
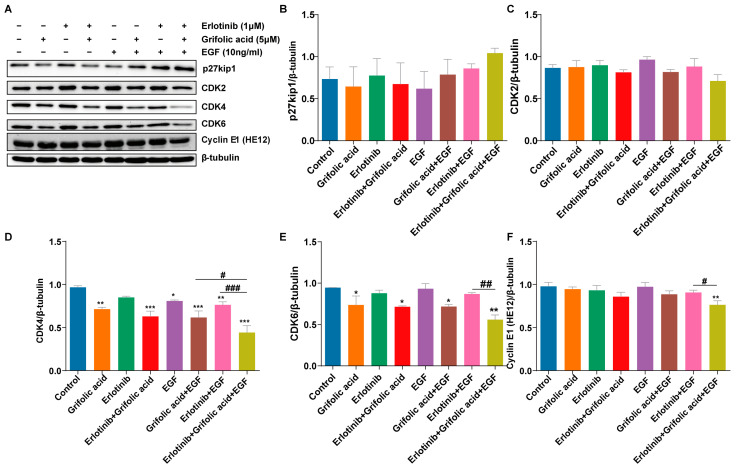
Cycle-related protein expression. The ANCI-H1781 cells were treated with erlotinib (1 μM), grifolic acid (5 μM), and EGF (10 ng/mL) for 24 h. The cycle-related protein-related proteins were assessed by immunoblot. β-tubulin was used as a loading control. The experiments were repeated at least three times. Quantitative analysis (**B**–**F**) of (**A**). The WB gels in Figure 6 were the cropping of the full-length gels. The full-length gels are presented in Supplementary Figure S4. The results were analyzed by one-way ANOVA with a significant difference versus combination-therapy group, ^#^ 0.01 ≤ *p* < 0.05, ^##^ 0.001 ≤ *p* < 0.01, ^###^ *p* < 0.001. There was a significant difference versus control, * 0.01 ≤ *p* < 0.05, ** 0.001 ≤ *p* < 0.01, *** *p* < 0.001.

To further determine why the combination of grifolic acid and erlotinib caused synergistic inhibition of cell growth, we investigated the cell cycle distribution. We further evaluated the effects of the drug combination by examining the levels of p27, CDK2, CDK4, CDK6, and Cyclin E1, each of which plays a role in cell cycle regulation. Treatment with grifolic acid alone had weak effects on the levels of these proteins in the NCI-H1781 cells. However, the administration of grifolic acid and erlotinib together led to decreased expression of CDK4, CDK6, and Cyclin E1 (HE12) and increased expression levels of p27Kip1 (not significant) (Figure 6A–F). These results confirm that in the presence of EGF, the synergistic combination of grifolic acid and erlotinib enhances cell cycle arrest.

## 3. Materials and Methods

### 3.1. Reagents

3-(4,5-dimethyl-2-thiazolyl)-2,5-diphenyl tetrazolium bromide (MTT) was obtained from Sigma–Aldrich (M2128, Louis, MO, USA). EGF was obtained from Sino Biological (10605-HNAE, Beijing, China). All compounds were purchased from BioBioPha (Kunming, Yunnan, China). Epidermal growth factor receptor tyrosine kinase inhibitors (EGFR-TKIs) were purchased from Meilunbio, including gefitinib (MB1112, Dalian, Liaoning, China), lapatinib (MB1463), and erlotinib (MB1734).

### 3.2. Protein–Protein Interaction (PPI) Network Analysis

The intersection targets were uploaded to the String database (version 12.0) (https://cn.string-db.org/, accessed on 1 January 2022) to generate the PPI network as “Homo sapiens”. The results were visualized using CytoScape 3.9.1 software. The CytoNCA too (v2.1.6) was used to analyze the network topology parameters; the larger the area of the nodes in the figure, the darker the color, the greater their influence.

### 3.3. GO Function of Target and KEGG Pathway Enrichment Analysis

The cluster Profiler R software package (v4.0) was used to perform GO and KEGG enrichment analysis for intersection targets, and GO analysis was completed from three aspects: biological process (BP), molecular function (MF), and cellular component (CC). The results were screened with *p* ≤ 0.01 and visualized with the “ggplot2” R package.

### 3.4. Molecular Docking Study

Molecular docking was carried out with AutoDock (v1.5.7). The structure of grifolic acid (CAS: 80557-12-6) from Chem 3D and the structure of EGFR (PDB ID 3njp) was obtained from the Protein Data Bank (http://www.rcsb.org/, accessed on 1 January 2021). The docking parameters were set to their default values, the number of genetic algorithm runs was set to 50, the and maximum number of evals (medium) was set to 3,000,000. Conformation was selected for visual analysis using PyMol (v4.6). Mode of action analyses were performed using Discovery Studio visualization software (v4.5).

### 3.5. Cell Culture

Human non-small cell lung cancer cells, NCI-H1975, human bronchiolar alveolar cells, NCI-H1781, human lung adenocarcinoma epithelial cells, and NCI-H441 were cultured in RPMI 1640 (MA0546, meilunbio), while human epidermal cancer cells A431 were cultured in DMEM medium (MA0545, meilunbio). The media were supplied with 10% fetal bovine serum (BSA, 04-010-1A, Biological Industries, Beit Haemek, Israel), whereas cells were grown in a 5% CO_2_ incubator at 37 °C.

### 3.6. Cell Viability Assay

NCI-H1975, NCI-H441, and NCI-H1781 cells were obtained from the Kunming Cell Bank of the Chinese Academy of Sciences (Kunming, China). The cells (3 × 10^4^/well) were seeded in a 96-well plate, with 200 μL media in each well. After overnight, NCI-H1975, NCI-H441, and NCI-H1781 cells were treated with EGFR-TKIs (gefitinib, lapatinib and erlotinib,1 μM), EGF (10 ng/mL), or compounds (10 μM) for 48 h. A431 cells were treated with inhibitors (gefitinib, lapatinib and erlotinib, 0.01 μM), EGF (10 ng/mL), or compounds (10 μM) for 48 h. The MTT assay was then carried out to assess the effect of compounds combined with three inhibitors on cell viability. The optical density of each well was measured at 492 nm by using a Flex Station 3 MultiMode Microplate Reader (Molecular Devices, San Jose, CA, USA).

In the concentration screening experiment, the NCI-H1781 cells were treated with different doses of grifolic acid for 48 h. The MTT assay was then carried out to assess the effect of different doses of grifolic acid on cell viability.

### 3.7. Colony Formation Assay

NCI-H1781 cells (5 × 10^3^ cells/plate) were treated with grifolic acid (5 μM), erlotinib (1 μM), or grifolic acid and erlotinib in a 6-well plate for 10 days. The cells were fixed in 4% paraformaldehyde, stained with 0.1% crystal violet, rinsed with water, dried, and photographed. The crystal violet was dissolved in 5% sodium dodecyl sulfate solution, and absorbance was detected at 570 nm.

### 3.8. Western Blotting

NCI-H1781 cells (2.5 × 10^6^) were inoculated into a Petri dish with a diameter of 60 mm and cultured overnight. Then, NCI-H1781 cells were cultured in serum-free RPMI 1640 medium for 16 h. Subsequently, the cells were treated with erlotinib (1 μM) for 1 h and treated with grifolic acid (5 μM) and EGF (10 ng/mL) for 30 min. The cells were first lysed in RIPA buffer containing PMSF (R0010, Solarbio, Beijing, China). Protein assay was then used to quantify the concentration using a BCA Protein Assay Kit (P0011, Beyotime, Shanghai, China). Proteins (50 μg) from each lysate sample were then separated by SDS-PAGE and then transferred to polyvinylidene difluoride (PVDF) membrane (Millipore, Merck KGaA, Darmstadt, Germany). The membranes were blocked in 5% of skill milk buffer for 1 h and then incubated with primary antibodies overnight at 4 °C, including β-tubulin, EGF receptor, phospho-EGF receptor (Tyr1068), AKT, phospho-AKT, p44/42 MAPK (ERK1/2), phospho-p44/42 MAPK (ERK1/2) (Thr202/Tyr204), Cleaved PARP, CDK2, CDK4, CDK6, Cyclin E1 (HE12), and p27Kip1 and Bcl-2 antibodies (1:1000, all from Cell Signaling Technology, Beverly, MA, USA). They were used in combination with horseradish peroxidase-labeled anti-rabbit (CTS005, R&D Systems, Nasdaq, MN, USA) or anti-mouse (CTS002, R&D Systems) IgG, and the target proteins were detected using chemiluminescence (Catalog No. 4AW011-500, 4A Biotech Co., Ltd., Beijing, China).

### 3.9. Statistical Analysis

The data are presented as the mean ± standard error of the mean (SEM). The differences between groups were examined by one-way ANOVA, with post hoc LSD’s multiple comparison tests, using SPSS software (v23, International Business Machines, Armonk, NY, USA). A *p*-value of <0.05 was considered to indicate statistical significance. Western blotting was analyzed using Image J software (v1.8.0, National Institutes of Health, Bethesda, MD, USA).

## 4. Discussion

Lung cancer is the leading cause of cancer-related death in the world, with a predicted 5-year survival rate of 15.9%, a figure that has just marginally improved over the past few decades [21]. Lung cancer is typically silent in the early stages. When symptoms emerge, they are usually nonspecific, such as difficulty breathing and coughing, and are often regarded by patients as being a part of aging, or in association with their smoking history. The result is that lung cancer is usually not diagnosed until later in the disease. The emergence of effective targeted therapies in NSCLC has driven significant improvements in survival.

EGFR is vital to lung cancers in pathogenesis and disease progression, and it is aberrantly expressed in NSCLC. There are two types of anti-EGFR drugs: monoclonal antibodies and tyrosine kinase inhibitors. EGFR-targeted therapy has achieved good effects in NSCLC, but only a small fraction of patients benefit from it. Unfortunately, after a period of treatment, patients will acquire drug resistance, underlining the necessity for alternative strategies to suppress EGFR pathway [11,12]. A recent study has shown that facilitating EGFR degradation is a potential therapeutic treatment in NSCLC, but no such medicine is available clinically [22]. EGFR signaling pathways remain the major molecule targets for the identification and exploitation of natural drugs that are effective in the treatment of NSCLC. Thus, it is vital to look for a novel, natural compound for the discovery of new methods of treatment, including complementary therapies that can be used in combination with existing treatments to reduce doses of toxic drugs and overcome drug resistance.

**Figure 7:**
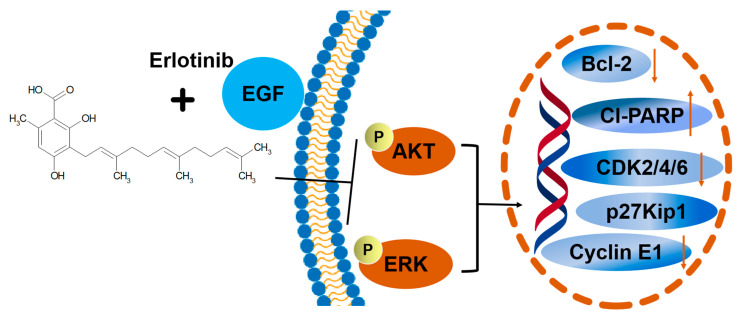
Schematic model of the molecular mechanism.

Natural compounds derived from plants are used for medical purposes throughout the world. In the current study, we investigated natural compounds’ potential utility in the treatment of NCI-H1781 cells. In this study, 168 direct target genes of 17 compounds were identified for NSCLC. GO enrichment analysis suggested that compounds were involved in the main biological processes, including protein phosphorylation, positive regulation of cell proliferation, and negative regulation of the apoptotic process. The enrichment analysis of KEGG pathway suggested that compounds had therapeutic effect on NSCLC by regulating pathways, including PI3K-Akt signaling pathway and erbB signaling pathway (Figure 1). Through screening 17 natural compounds, we identified a natural product, grifolic acid, as a potential anti-tumor drug (Table S1, Figure 2, Supplementary S1). The study of grifolic acid has focused on FFAR. Although it has anticancer activity, there are no studies of grifolic acid in lung cancer research [18,19,20]. Molecular docking simulation results have represented that grifolic acid and EGFR can dock well, and the binding energy was −4.1 kcal/mol. Grifolic acid forms 5 hydrogen bonds with EGFR residues, and Pro-219 is involved in the formation of hydrophobic interactions (Figure 3). So, this study was designed to investigate whether grifolic acid combined with low-dose erlotinib could inhibit NCI-H1781 cell proliferation and EGFR signaling. It is well-known that EGFR ligand leads to autophosphorylation and downstream signaling molecule activation [6]. The results of our study have revealed that in the presence of the ligand-EGF, combination therapy inhibits the levels of pEGFR and phosphorylated downstream target kinases ERK1/2 and AKT in NCI-H1781 cells (Figure 4 and Figure 7). Bcl-2, an anti-apoptotic proto-oncogene, is highly expression in NSCLC (37% of cases) and plays a role in cancer cell apoptosis. Among them, squamous cell carcinoma cases account for 25%, while adenocarcinoma cases make up 12% [23]. Our data revealed that combination therapy decreases the protein level of Bcl-2 and enhances the expression of cleaved-PARP (Figure 5 and Figure 7).

One of the important characteristics of malignant tumors is uncontrolled proliferation. Cyclin-dependent kinase (CDK) is a type of serine/threonine protein kinase, and it is also a key regulator for cell cycle regulation [24]. The CDK overexpression can result in tumor progression and uncontrolled proliferation [25]. Previously, a study demonstrated that cyclin E1 and D protein overexpression may be a significant part of the multistep process of lung tumorigenesis [26]. Simultaneously, cyclin E-CDK2 complexes phosphorylate a variety of proteins required for cell cycle progression, DNA replication, and centrosome replication [27]. CDK 4 and 6 interacted with D-type cyclins to phosphorylate substrates and promote G1 to S phase cell cycle progression in cancer cells [28,29]. Clinical trials have shown that abemaciclib, a CDK4/6 inhibitor, has anti-tumor activity in breast cancer, lung cancer, and other solid tumors [29,30]. So, suppressing CDK4/6 expression for patients is vital. p27Kip1 is an atypical tumor suppressor, which belongs to the Cip/Kip family of CDK inhibitors. p27Kip1 regulates the activity of cyclin-dependent kinases to control G0 to S phase transitions [31,32]. In the current study, we demonstrated that grifolic acid combined with erlotinib reduces the expression of cyclinE, CDK4, and CDK6 proteins and promotes the expression of the pro-apoptotic protein p27Kip1 (not significant) (Figure 6 and Figure 7). Although grifolic acid had a good inhibitory effect on NCI-H1781 cells, the current study suggests that the grifolic acid may inhibit the activation of EGFR through binding with the extracellular domain of EGFR. The correlation between EGFR and grifolic acid will be elucidated in our subsequent investigations. Additionally, because there are very few in vivo studies on the pharmacological activities of grifolic acid, its effect on anti-tumor effect in vivo has not yet been verified. The above unsolved issues are worthy of further investigation in the future.

## 5. Conclusions

In conclusion, we mainly focused on targeting EGFR signaling and found grifolic acid to be potent against the WT-EGFR in a drug-screening protocol, confirming its activity in in vitro assays. This is the first study to indicate the anticancer activity of grifolic acid in NSCLC. Particularly, combination therapy causes cell cycle arrest and apoptosis. Meanwhile, it also supports the important idea that grifolic acid is a potential drug for treating lung cancer. Our results provide insight into the application of grifolic acid for WT-EGFR NSCLC therapy.

## Figures and Tables

**Table 1:** Detailed interactions of grifolic acid–EGFR.

System	Donor	Accepter	Distance (Å)	Types
EGFR-grifolic acid	grifolic acid C=O	Ser196 (Chain B)	3.9	Hydrogen bond
	grifolic acid H	Cys208 (Chain B)	4.8	Hydrogen bond
	grifolic acid H	Cys208 (Chain B)	4.3	Hydrogen bond
	grifolic acid C	Cys195 (Chain B)	4.7	Carbon Hydrogen bond
	grifolic acid C	His209 (Chain B)	4.9	Carbon Hydrogen bond
	grifolic acid C	Pro219 (Chain A)	4.9	Hydrophobic (Alkyl)
	grifolic acid C	Pro219 (Chain A)	4.9	Hydrophobic (Alkyl)
	grifolic acid C	Pro219 (Chain A)	3.5	Hydrophobic (Alkyl)
	grifolic acid C	Pro219 (Chain A)	4.5	Hydrophobic (Alkyl)
	grifolic acid C	Pro219 (Chain B)	4.2	Hydrophobic (Alkyl)

## Data Availability

All data of this study are included within the article and its Supplemental Information and are available from the corresponding author upon reasonable request.

## Supplementary Materials

The following supporting information can be downloaded at: https://www.mdpi.com/article/10.3390/ijms26020629/s1.

## Abbreviations

EGFR	epidermal growth factor receptor
NSCLC	non-small cell lung cancer
TKI	tyrosine kinase inhibitors
MTT	3-(4,5-dimethyl-2-thiazolyl)-2,5-diphenyl tetrazolium bromide;
WT	wild-type
