# Supplementary material for: Network Pharmacology Approach and Experimental Verification to Explore the Anti-NSCLC Mechanism of Grifolic Acid
Source: Int J Mol Sci. 2025 Jan 13;26(2):629. doi: 10.3390/ijms26020629 (PMC11765843; doi:10.3390/ijms26020629)
Supplement: Supplementary file 1 [file ijms-26-00629-s001.zip › ijms-3349948-supplementary.pdf]

A

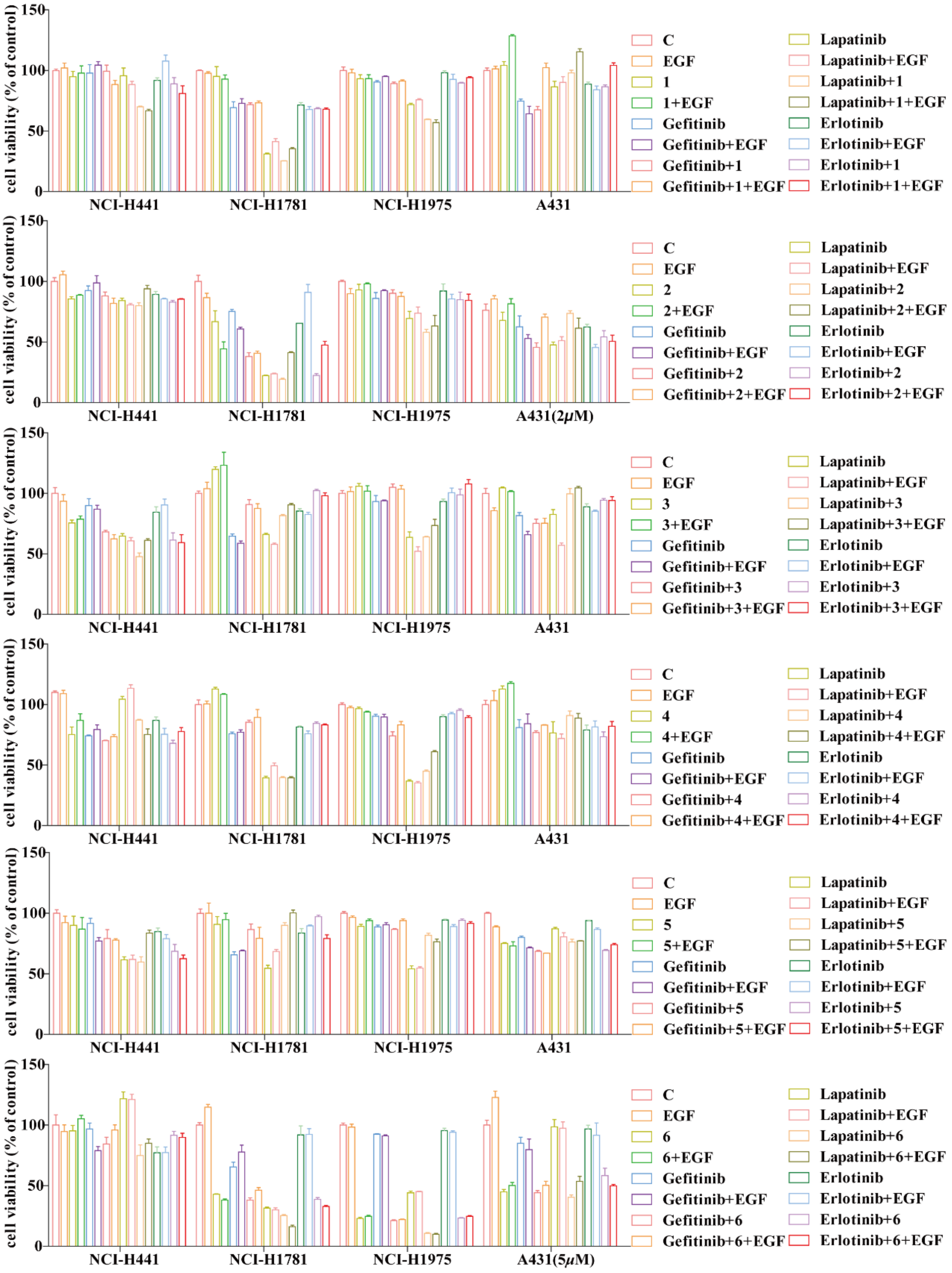

cell viability (% of control)
150
100
50
0
NCI-H441
NCI-H1781
NCI-H1975
A431
C
EGF
1
1+EGF
Gefitinib
Gefitinib+EGF
Gefitinib+1
Gefitinib+1+EGF
Lapatinib
Lapatinib+EGF
Lapatinib+1
Lapatinib+1+EGF
Erlotinib
Erlotinib+EGF
Erlotinib+1
Erlotinib+1+EGF
A431(2μM)
2
2+EGF
Gefitinib+2
Gefitinib+2+EGF
Lapatinib+2
Lapatinib+2+EGF
Erlotinib+2
Erlotinib+2+EGF
3
3+EGF
Gefitinib+3
Gefitinib+3+EGF
Lapatinib+3
Lapatinib+3+EGF
Erlotinib+3
Erlotinib+3+EGF
4
4+EGF
Gefitinib+4
Gefitinib+4+EGF
Lapatinib+4
Lapatinib+4+EGF
Erlotinib+4
Erlotinib+4+EGF
5
5+EGF
Gefitinib+5
Gefitinib+5+EGF
Lapatinib+5
Lapatinib+5+EGF
Erlotinib+5
Erlotinib+5+EGF
6
6+EGF
Gefitinib+6
Gefitinib+6+EGF
Lapatinib+6
Lapatinib+6+EGF
Erlotinib+6
Erlotinib+6+EGF
A431(5μM)

B

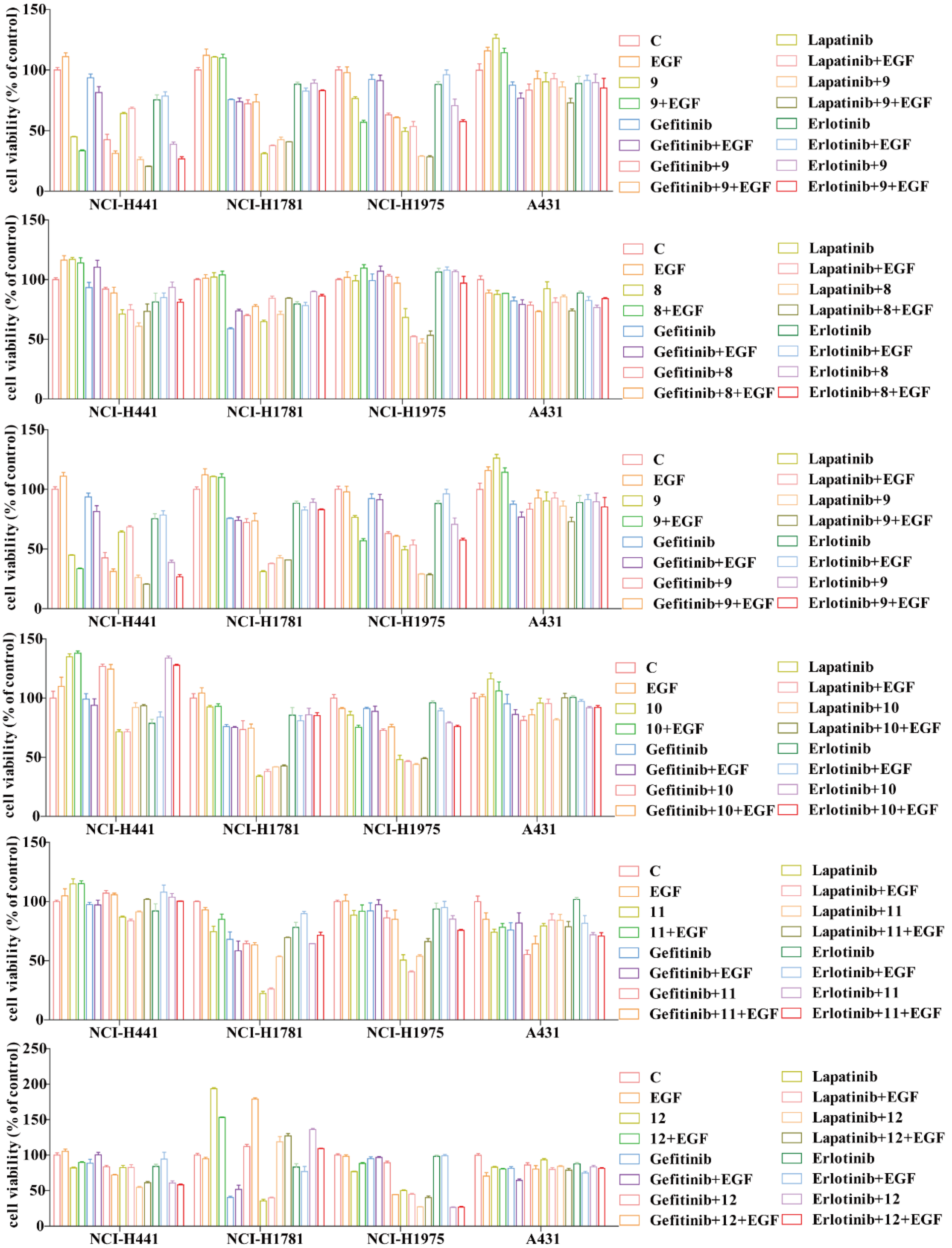
cell viability (% of control)
0
50
100
150
NCI-H441
NCI-H1781
NCI-H1975
A431
C
EGF
9
9+EGF
Gefitinib
Gefitinib+EGF
Gefitinib+9
Gefitinib+9+EGF
Lapatinib
Lapatinib+EGF
Lapatinib+9
Lapatinib+9+EGF
Erlotinib
Erlotinib+EGF
Erlotinib+9
Erlotinib+9+EGF
cell viability (% of control)
0
50
100
150
NCI-H441
NCI-H1781
NCI-H1975
A431
C
EGF
8
8+EGF
Gefitinib
Gefitinib+EGF
Gefitinib+8
Gefitinib+8+EGF
Lapatinib
Lapatinib+EGF
Lapatinib+8
Lapatinib+8+EGF
Erlotinib
Erlotinib+EGF
Erlotinib+8
Erlotinib+8+EGF
cell viability (% of control)
0
50
100
150
NCI-H441
NCI-H1781
NCI-H1975
A431
C
EGF
9
9+EGF
Gefitinib
Gefitinib+EGF
Gefitinib+9
Gefitinib+9+EGF
Lapatinib
Lapatinib+EGF
Lapatinib+9
Lapatinib+9+EGF
Erlotinib
Erlotinib+EGF
Erlotinib+9
Erlotinib+9+EGF
cell viability (% of control)
0
50
100
150
NCI-H441
NCI-H1781
NCI-H1975
A431
C
EGF
10
10+EGF
Gefitinib
Gefitinib+EGF
Gefitinib+10
Gefitinib+10+EGF
Lapatinib
Lapatinib+EGF
Lapatinib+10
Lapatinib+10+EGF
Erlotinib
Erlotinib+EGF
Erlotinib+10
Erlotinib+10+EGF
cell viability (% of control)
0
50
100
150
NCI-H441
NCI-H1781
NCI-H1975
A431
C
EGF
11
11+EGF
Gefitinib
Gefitinib+EGF
Gefitinib+11
Gefitinib+11+EGF
Lapatinib
Lapatinib+EGF
Lapatinib+11
Lapatinib+11+EGF
Erlotinib
Erlotinib+EGF
Erlotinib+11
Erlotinib+11+EGF
cell viability (% of control)
0
50
100
150
200
250
NCI-H441
NCI-H1781
NCI-H1975
A431
C
EGF
12
12+EGF
Gefitinib
Gefitinib+EGF
Gefitinib+12
Gefitinib+12+EGF
Lapatinib
Lapatinib+EGF
Lapatinib+12
Lapatinib+12+EGF
Erlotinib
Erlotinib+EGF
Erlotinib+12
Erlotinib+12+EGF

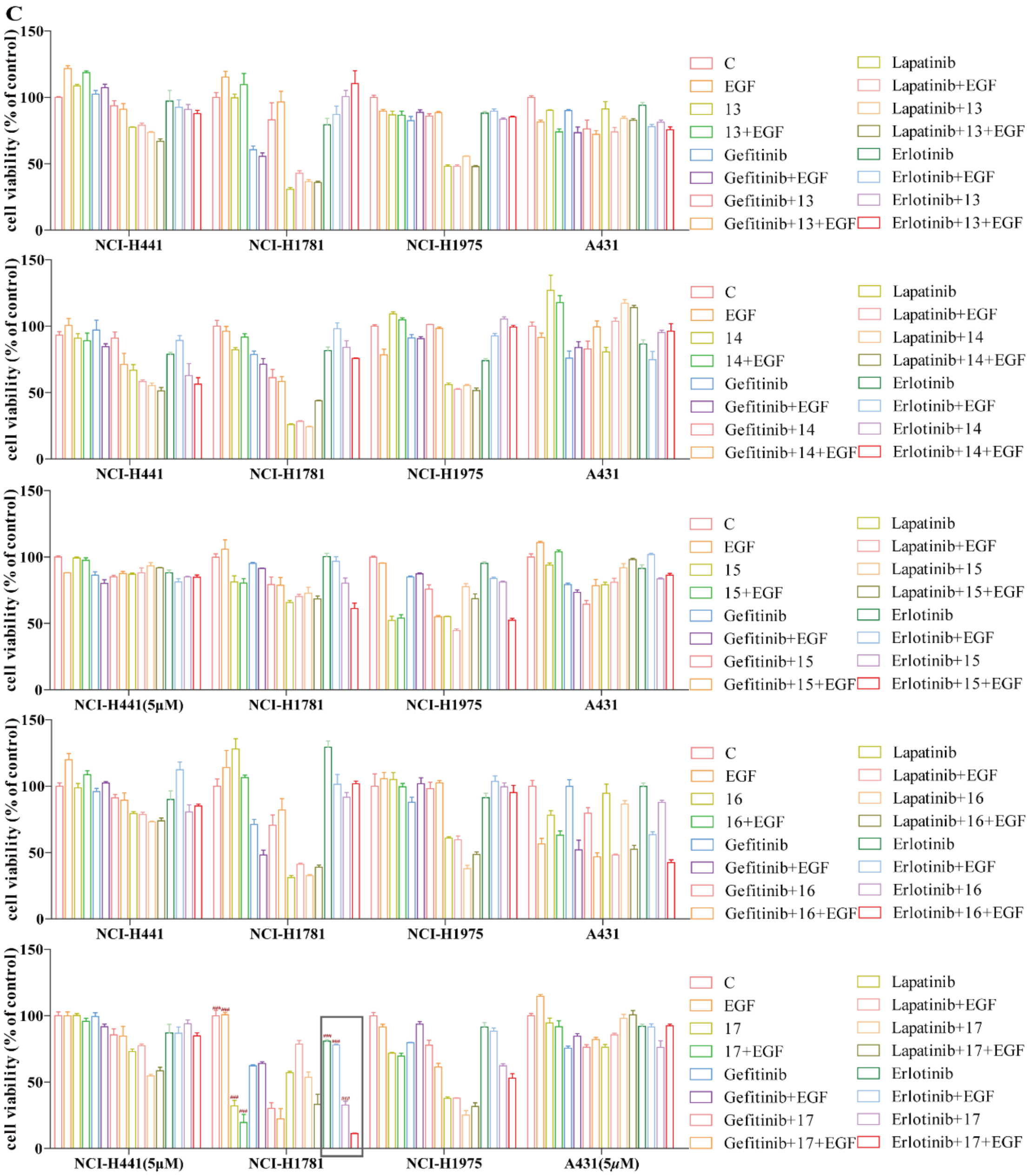

Figure S1. Cell viability assay. MTT screened 17 compounds and 3 EGFR-TKIs to determine the activity of 4 cells in the presence of EGF, each picture represents the screening results of one compound, 3 EGFR-TKIs and 4 cells. Default without special instructions the concentration of the compound is 10μM. Results were analysis by one-way ANOVA, significant difference versus combination-therapy group, $^{\#}$ 0.01 ≤ P<0.05, $^{\#\#}$ 0.001 ≤ P<0.01, $^{\#\#\#}$ P<0.001.

Table S1 The name, CAS number, and chemical structure of these 17 compounds.

ID	Name of Substance	CAS No.	Structure
1	Eichlerianic acid	56421-13-7	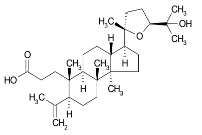
2	Richenoic acid	134476-74-7	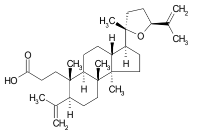
3	3-Epicorosolic acid	52213-27-1	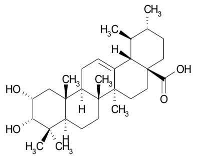
4	15,16-Epoxy-12-hydroxylabda-8(17),13(16),14-triene	61597-55-5	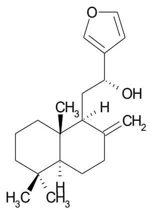
5	Isogarciniaxanthone E	659747-28-1	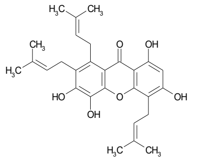
6	Walsuralactam A	1370556-82-3	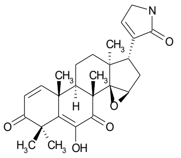
7	Luteone	41743-56-0	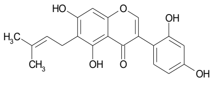
8	2,2',3'-Trihydroxy-4,6-dimethoxybenzophenone	219861-73-1	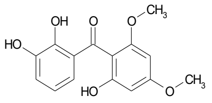
9	Coronarin D	119188-37-3	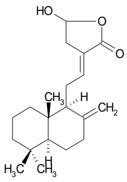

ID	Name of Substance	CAS No.	Structure
10	Magnolol	528-43-8	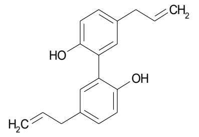
11	Isobavachin	31524-62-6	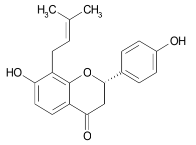
12	Griffipavixanthone	219649-95-3	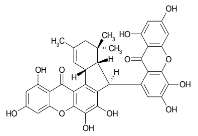
13	Nobiletin	478-01-3	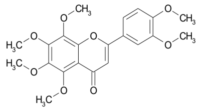
14	Triptotriterpenic acid A	84108-17-8	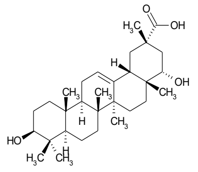
15	Simonsino	155709-40-3	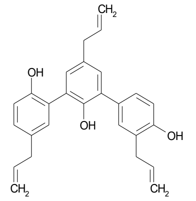
16	4-Hydroxycinnamamid	194940-15-3	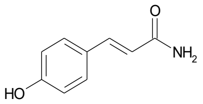
17	Grifolic acid	80557-12-6	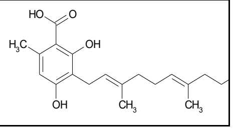

Table S2 Molecular docking binding energy of 17 compounds and targets (Kcal/mol).

Ligands	Binding force (Kcal/mol)
Eichlerianic acid	-5.95
Richenoic acid	-6.58
3-Epicorosolic acid	-6.42
15,16-Epoxy-12-hydroxylabda-8(17),13(16),14-triene	-6.55
Isogarciniaxanthone E	-4.56
Walsuralactam A	-7.81
Luteone	-5.15
2,2',3'-Trihydroxy-4,6-dimethoxybenzophenone	-4.39
Coronarin D	-6.4
Magnolol	-5.31
Isobavachin	-6.52
Griffipavixanthone	-6.57
Nobiletin	-4.41
Triptotriterpenic acid A	-5.96
Simonsino	-4.90
4-Hydroxycinnamamid	-7.61
Grifolic acid	-4.10
